# Introduction and Spatial–Temporal Distribution of Oropouche Virus in Rio de Janeiro State, Brazil

**DOI:** 10.3390/pathogens14080833

**Published:** 2025-08-21

**Authors:** Fábio Burack da Costa, Andrea Cony Cavalcanti, Rafael Santos Erbisti, Vanessa Zaquieu Dias, Cristiane Gomes de Castro Moreira, Mateus Marques Grifo, Maria Carmelita dos Santos Vaz, Adriana Cardoso Camargo, Leandro Magalhães de Souza, Flávia Barreto dos Santos, Mário Sérgio Ribeiro, Viviana Malirat, Nildimar Alves Honório, Renata Campos Azevedo

**Affiliations:** 1 Laboratório das Interações Vírus-Hospedeiros—LIVH, Instituto Oswaldo Cruz, Fiocruz, Rio de Janeiro CEP 21040-360, RJ, Brazil; fabio_burack@hotmail.com (F.B.d.C.); flaviab@ioc.fiocruz.br (F.B.d.S.); 2 Departamento de Virologia, Instituto de Microbiologia Paulo de Góes, Universidade Federal do Rio de Janeiro, Rio de Janeiro CEP 21941-902, RJ, Brazil; andreacony84@gmail.com (A.C.C.); vanessazaquieu@micro.ufrj.br (V.Z.D.); crisarthurpharma@gmail.com (C.G.d.C.M.); m.marchio2002@gmail.com (M.M.G.); 3 Laboratório Central de Saúde Pública Noel Nutels—LACEN-RJ, Rio de Janeiro CEP 20231-092, RJ, Brazil; mcarmelitavaz@yahoo.com.br (M.C.d.S.V.); adrianaccamargo14@gmail.com (A.C.C.); magalhaessouza@yahoo.com (L.M.d.S.); 4 Departamento de Estatística, Universidade Federal Fluminense, Niterói CEP 24210-201, RJ, Brazil; rerbisti@id.uff.br; 5 Secretaria Estadual de Saúde do Rio de Janeiro—SES/RJ, Rio de Janeiro CEP 20261-901, RJ, Brazil; mario_sesrj@yahoo.com.br; 6 Centro de Virología Humana y Animal (CEVHAN), Consejo Nacional de Investigaciones Científicas y Técnicas (CONICET), Universidad Abierta Interamericana (UAI), Buenos Aires CP1270, Argentina; vmalirat@hotmail.com; 7 Núcleo Operacional Sentinela de Mosquitos Vetores-Nosmove-Fiocruz, Rio de Janeiro CEP 21040-360, RJ, Brazil

**Keywords:** Oropouche virus, *Orthobunyavirus*, emergence, Rio de Janeiro

## Abstract

The Oropouche virus (OROV) has been circulating in the Amazon region since the 1960s, with a progressive increase in outbreaks and human cases reported in Brazil and neighboring countries. In the Rio de Janeiro state, there has been a significant rise in suspected cases of arboviruses, with only 30% of laboratory tests confirming infections with dengue, Zika, or chikungunya viruses. The investigation of OROV virus circulation in the Rio de Janeiro state was initiated and confirmed in April 2024. Our study aimed to retrospectively investigate OROV infections in dengue-suspected cases with inconclusive diagnosis in order to better understand the temporal and geographic introduction of OROV in the Rio de Janeiro state. Municipalities from Rio de Janeiro with arbovirus-like fever cases but a low percentage of dengue-positive RT-PCR test confirmations were identified in the laboratory database. Samples were selected for testing OROV infections using real-time RT-PCR as recommended by the Brazilian Ministry of Health. Municipalities in the Middle Paraíba region of the state showed 93% negative tests results for dengue, Zika, and chikungunya starting in September 2023. A total of 118 positive cases of Oropouche were recorded in the state of Rio de Janeiro between March and July 2024. Moreover, by genome sequencing of eight strains, it was shown that OROV circulating in Rio de Janeiro belongs to recently emergent M_1_L_2_S_2_ lineage. Our findings retrospectively revealed a concentration of cases in the Middle Paraíba region and an outbreak in the rural village of Cacaria, located in the municipality of Piraí. According to our data, this region is the first area with sustained transmission in the Rio de Janeiro state.

## 1. Introduction

Oropouche fever is a viral disease characterized by headache, myalgia, arthralgia, anorexia, dizziness, and photophobia. In some cases, a rubella-like skin rash may occur. The disease can present two symptomatic phases, with a second, generally milder wave of symptoms occurring 2 to 3 weeks after the initial episode [1]. Oropouche virus (OROV) was first isolated from a febrile patient in Trinidad and Tobago in 1955, being associated with Oropouche fever, and then isolated from mosquitoes of the species *Coquillettidia* (*Rhynchotaenia*) *venezuelensis* (Theobald, 1912), pointing to the possibility of vector-borne transmission [2].

The etiological agent of Oropouche fever is OROV, belonging to the family *Peribunyaviridae*, species *Orthobunyavirus oropoucheense*, which currently includes four prototype strains: Oropouche virus, Iquitos virus, Madre de Dios virus, and Perdões virus (available at ICTV https://ictv.global/report/chapter/peribunyaviridae accessed on 17 October 2024). Members of the *Orthobunyavirus* genus are enveloped and have a segmented RNA genome of negative polarity, which allows for high genetic variability due to reassortment processes [3]. The genome is divided into three segments: the L segment encodes the large viral RNA polymerase/endonuclease; the M segment encodes the surface glycoproteins Gn and Gc and the non-structural protein NSm; and the S segment encodes the nucleocapsid protein N and the non-structural protein NSs [4]. Recent phylogenetic analyses have revealed two major viral lineages for the M (M1 and M2) and L (L1 and L2) segments, while the S segment presents three major lineages (S1, S2, and S3) along with several minor lineages (Sx) [5].

The first recorded cases of OROV infecting humans in Brazil date back to 1960, during the construction of the Belém–Brasília road [6]. In addition, the virus was first isolated from the blood of a sloth (*Bradypus tridactylus*) and *Ochlerotatus serratus* (Theobald, 1901) mosquitoes captured in the Amazon region, confirming its wild circulation. Afterwards, outbreaks were recorded in the 1960s, 1970s and 1980s in northern Brazilian states, and biting midge *Culicoides paraensis* was associated as the primary vector in urban/rural areas [4]. Given the significant overlap in signs and symptoms between Oropouche fever and other arboviral infections, the disease is likely underreported. By 2018, the presence of the virus had been reported in all regions of the country [7]. As described by Naveca et al. (2024), the strain that recently spread throughout Brazil is an M_1_L_2_S_2_ reassortant originated from an M_2_L_2_S_2_ virus that circulated in Peru, Colombia, and Ecuador from 2008 to 2021 and an M_1_L_2_S_3_ virus that circulated in the eastern Amazon between 2008 and 2018 [5].

From September 2023 to July 2024, the state of Rio de Janeiro reported a higher number of suspected dengue cases. According to the State Department of Health (SES/RJ), 423,084 suspected cases were notified and 86,367 were subjected to laboratory analysis for dengue virus (DENV), Zika virus (ZIKV), and chikungunya virus (CHIKV), with DENV-2 and DENV-1 being confirmed in only 30% of them [8].

Due to the increase in OROV infections registered in 2023 in the North region of Brazil, the Central Public Health Laboratory of Rio de Janeiro (LACEN/RJ) began testing dengue-negative cases with a duplex test for OROV and Mayaro virus (MAYV) in April 2024, following the Brazilian Ministry of Health recommendation. OROV was first detected in patients from the Middle Paraíba region, confirming autochthonous circulation in the Rio de Janeiro state. Therefore, to better understand the dynamics of OROV circulation in Rio de Janeiro, our study aimed to retrospectively investigate the presence of OROV infection in dengue-suspected cases with an inconclusive diagnosis.

## 2. Materials and Methods

### 2.1. Study Area

The Rio de Janeiro state is located in the Southeastern region of Brazil with approximately 16,055,174 inhabitants, 43,750,425 km^2^, and 92 municipalities [9]. These municipalities are grouped into nine health regions: South Center Fluminense, Metropolitan I, Metropolitan II, Northwest, North, Mountain, Ilha Grande Bay, Coastal lowland, and Middle Paraíba (Supplementary Figure S1). The Metropolitan I and Metropolitan II regions have the highest population density in the state. The climate is tropical with rainfall and higher temperatures during the summer.

### 2.2. Study Population and Ethics Committee

Information regarding suspected and confirmed cases of DENV, ZIKV, and CHIKV from September 2023 to March 2024 was obtained from the National Laboratory database system (GAL) and analyzed to identify regions with a high number of suspected cases without confirmation of the etiological agent. Samples from the selected areas were tested for OROV and MAYV infection. From April onward, 10% of negative samples from DENV, ZIKV, and CHIKV were randomly selected to represent all health regions of the state. This work was previously approved by the Ethics Committee of Fundação Oswaldo Cruz, CEP: 6621940. The availability of samples for research purposes during outbreaks of national concern is permitted under the terms of Resolution 510/2016 of the National Ethical Committee for Research (CONEP), which authorizes the use of clinical samples collected in the Brazilian Central Public Health Laboratories without the necessity of informed consent to accelerate knowledge building and contribute to surveillance and outbreak response. All data were extracted from the database by authorized professionals, and the information was provided to the researchers in a coded format, ensuring anonymity.

### 2.3. Detection of Arboviruses by Real-Time RT-PCR and Nucleotide Sequencing

RNA was extracted from 200 µL of serum using the MagMAx viral pathogen (Thermo Fisher, Waltham, MA, USA) on the automated nucleic acid extractor King Fisher Apex device (Thermo Fisher, Waltham, MA, USA). The extracted RNA was then submitted to real-time RT-PCR using TaqMan probes (RT-qPCR) on QuantStudio 5 (Thermo Fisher, Waltham, MA, USA). For the molecular detection of DENV, ZIKV, and CHIKV, the Biomol ZDC kit (Ibmp, Paraná, Brazil) was used according to the manufacturer’s instructions. For the detection of OROV and MAYV, sets of primers and probes produced by Ibmp, Paraná, Brazil, were used as described by Naveca and cols [10], strictly following the amplification protocols. RT-PCR OROV-positive samples (*n* = 21) were selected for genome sequencing. Nested RT-PCR was conducted using oligonucleotides targeting the S, L, and M segments as described by Nascimento and cols [11]. Conventional RT-PCR was performed using Superscript III one-step RT-PCR kit (Invitrogen, Carlsbad, CA, USA), according to the manufacturer’s instructions. Cycle reactions were performed on MiniAmp Thermal Cyclers (Thermofisher, Waltham, MA, USA). Amplicons were visualized by 1.5% Agarose Gel Electrophoresis and quantified using NanoDrop™ One Microvolume UV-Vis Spectrophotometers (Thermo Scientific™). The nucleotide sequences were obtained by the Sanger technique, determined from 200 ng of amplicons, using the Big Dye Terminator kit 3.1 (Applied Biosystems, Waltham, MA, USA), following the manufacturer’s procedure and read in the ABI3730 genetic analyzer (Applied Biosystems, Waltham, MA, USA), following the manufacturer’s protocol. Raw sequence data were aligned, edited, and assembled using the BioEdit Sequence Alignment Editor, Version 7.0.5.3 [12].

### 2.4. Phylogenetic Analysis

Sequences of the three OROV genome segments available from GenBank at the National Center for Biotechnology Information (NCBI) were used for comparison. Samples used in the analysis (including those determined in this study) are listed in Supplementary Table S1 and cover historical samples as well as recently circulating OROV isolates. Alignments were compiled for an individual analysis of each of the three viral genome segments. Primers used covered regions between 56–621 nt, 2864–3564 nt, and 6–728 nt for L, M, and S segments, respectively. Positions were determined in comparison to OROV GenBank reference sequence NC_005776.1 (segment L), NC_005775.1 (segment M), and NC_005777.1 (segment S). Sequences were aligned using the Clustal X algorithm (version 2.1, https://evomics.org/resources/software/bioinformatics-software/clustal-x/ accessed on 10 August 2024), as implemented in the MEGA 11 software [13], and the alignments were manually checked and edited to ensure codon alignment. The molecular phylogeny was estimated for each alignment using the maximum-likelihood (ML) approach implemented in the MEGA package [14]. ML trees were estimated using a general time-reversible substitution model (GTR) with a gamma distribution model of among-site heterogeneity (5 categories). The GTR substitution model was chosen by means of the program MEGA, analyzing twenty-four different evolutionary models using Akaike Information Criteria (AIC) and a Likelihood Ratio Test (LRT) to identify the optimal evolutionary model. Codon positions included were 1st + 2nd + 3rd + Noncoding. All positions with less than 95% site coverage were eliminated (i.e., fewer than 5% alignment gaps, missing data, and ambiguous bases were allowed at any position; partial deletion option). Phylogenetic node support was assessed using a nonparametric bootstrap approach with 1000 replicates. To facilitate visualization, nodes were collapsed when needed.

### 2.5. Statistical Analysis

For statistical purposes, the requested tests for DENV, CHIKV, and ZIKV were aggregated into monthly temporal units and at the municipality spatial scale across the nine health regions. Additionally, suspected and confirmed cases of OROV were aggregated by epidemiological week for each municipality in the state of Rio de Janeiro. Summary measures were calculated for DENV, CHIKV, ZIKV, and OROV at both the municipal spatial scale and across the nine health regions. Thematic maps displaying the accumulated cases of OROV per month and municipality were created. The R software (version 4.3.1) [15] was used for the generation of maps, summary statistics, and graphics.

## 3. Results

**Figure 1:**
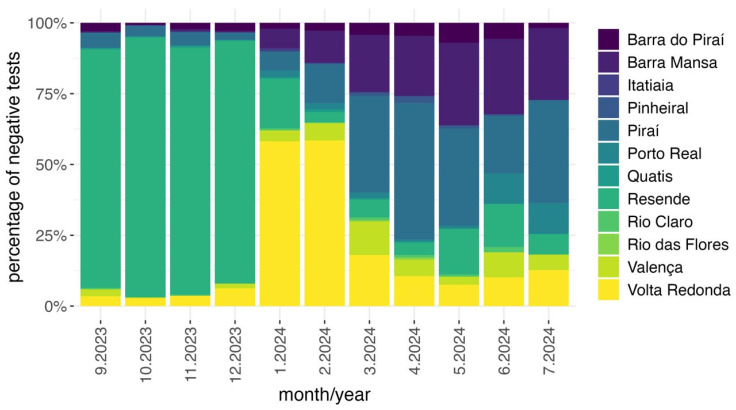
Percentage of negative tests for dengue, Zika, and chikungunya in the municipalities of the Middle Paraíba region, Rio de Janeiro state, from September 2023 to July 2024.

From September 2023 to July 2024 (35th epidemiological week of 2023 to 28th epidemiological week of 2024), a total of 86,367 real-time RT-PCR tests for ZIKV, DENV, and CHIKV (ZDC test) were conducted in the state of Rio de Janeiro, rendering 60,423 (70.0%) negative and 25,944 (30.0%) positive results (Supplementary Table S2). The first OROV case was detected in the Middle Paraíba region. Therefore, we analyzed the spatial–temporal distribution of RT-PCR-negative outcomes by municipality within this region. Figure 1 shows the percentage of ZDC-negative tests in the twelve municipalities for each month of the analysis period. From September to December 2023, the municipality of Resende had the highest number of tests conducted and, consequently, the highest number of negative results between January and February 2024. Volta Redonda accounted for nearly 60% of all negative ZDC results in the region. From March to July 2024, Barra Mansa and Piraí accounted for the highest numbers of negative results, 52.2% (467/893) and 50.2% (769/1533) tests, respectively. Together, these two municipalities represented 60.3% of all negative tests in the Middle Paraíba region (Figure 1).

To investigate possible introduction routes and understand the dynamics of OROV transmission in the state of Rio de Janeiro, we conducted a retrospective analysis focusing on the municipalities in Middle Paraíba alongside routine surveillance. Of the 1049 samples collected in Middle Paraíba, 76 were positive (Table 1). Among these, 71 were from Piraí, mainly in the village of Cacaria (22°42′49.4′′ S 43°50′36.4′′ W), a rural set in the Arara mountain area with approximately 1300 inhabitants, 974 households, of which only 152 do not have at least one facade facing the forest that borders the BR-101 road. The region is predominantly characterized by the Atlantic Forest biome and a tropical highland climate. The Cacaria outbreak began in March 2024 and extended until May 2024. Table 1 shows the number of suspected and positive cases tested by health regions over different periods. A total of 118 positive OROV cases were confirmed in the state of Rio de Janeiro. The Northwest region reported OROV cases only in April and May 2024, specifically in the municipality of Bom Jesus do Itabapoana, where seven positive cases were confirmed. Among the nine positive cases reported in the mountain region, six were in the Guapimirim municipality. The city of Rio de Janeiro, belonging to the Metropolitan I region, confirmed only seven positive cases of OROV.

**Figure 2:**
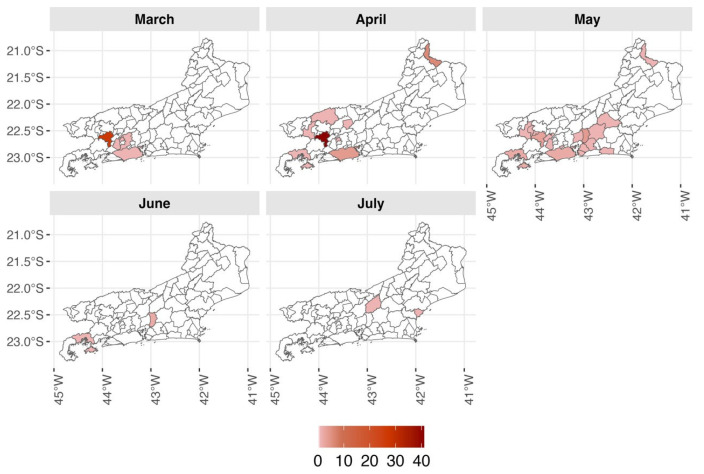
Positive cases of Oropouche virus, by municipality of Rio de Janeiro state, March to July 2024.

The spatial distribution of OROV-positive cases indicated that the Middle Paraíba and the Metropolitan regions were hotspot areas in March. Starting in April, a gradual expansion was observed towards the state’s interior and in the nearby coastal areas. In the following months, there was slight spatial dispersion, with positive cases being registered in almost all regions of the state, except for the North region. From June onward, a decrease in the number of positive cases was observed (Figure 2).

From the OROV-positive cases in the Rio de Janeiro state (*n* = 118), there was a total of 21 representatives of the municipalities across distinct regions presenting positive cases: Guapimirim and Nova Friburgo (Mountain); Barra Mansa, Volta Redonda, Piraí, and Valença (Middle Paraiba); Bom Jesus de Itabapoana (Northwest); Mesquita and Japeri (Metropolitan I), Saquarema (Coastal Lowland); Paracambi (South Center) and Angra dos Reis (Ilha Grande Bay) were selected for genome amplification. From those, all 21 had the L segment sequenced, in 14, the M segment sequences were obtained, and in 14, the S segment was also recovered. Overall, eight were successfully sequenced for all three genome segments. All sequences obtained in this study were deposited at Genbank under accession numbers PQ349278 to PQ349298 for the L segment; PQ349299 to PQ349312 for the M segment; and PQ295303 to PQ296316 for the S segment).

**Figure 3:**
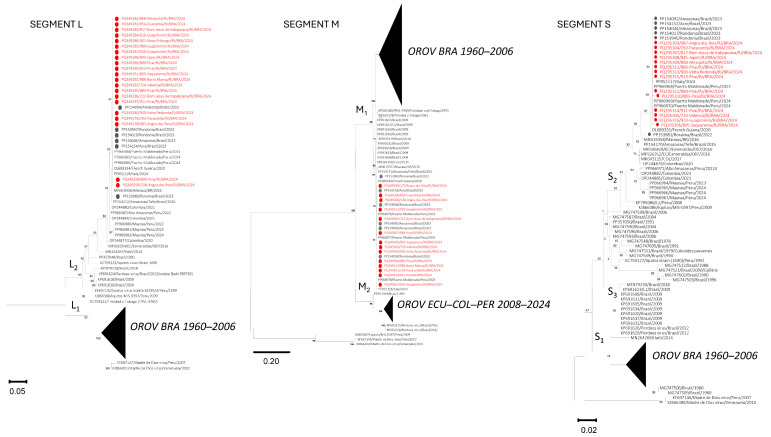
Phylogenetic analyses of the M, L, and S segments of OROV. Nucleotide sequences obtained in this study are highlighted in red and marked with a red dot. Closely related isolates (samples collected in the Northern region of Brazil from 2022 to 2024) are marked with a gray dot. The phylogenetic analysis of OROV sequences from segments L, M, and S (as indicated) was inferred by using the maximum-likelihood method and the general time-reversible model, as described in the Section 2. The genetic distance scale is indicated at the bottom of each panel. The percentage of trees on which the associated taxa clustered together is shown next to the branches. Evolutionary analyses were conducted in MEGA 11. To improve visualization, nodes with a large number of sequences were collapsed and indicated in the figure by a black triangle: OROV BRA 19602006: Isolates of OROV collected in Brazil from 1960 to 2006. OROV ECU–COL–PER 2008–2024: Isolates of OROV collected in Colombia, Ecuador, and Peru from 2008 to 2024.

A high nucleotide similarity (>99%) was observed among all sequences from the same genomic segment obtained for the different isolates analyzed in this study. The corresponding identity matrices are provided in Supplementary Material S1. A BLASTn search of all three genomic segments revealed that the sequences from Rio de Janeiro shared over 99% nucleotide identity with more than 100 sequences from outbreaks, originating primarily from other Brazilian states during the same year. Moreover, the phylogenetic analysis clustered the OROV strains from the Rio de Janeiro state together with sequences previously classified within lineages M_1_, L_2,_ and S_2_ (Figure 3). The genomic constellation identified in the Rio de Janeiro isolates matches with samples collected in the Northern region of Brazil between 2022 and 2024, reinforcing the evidence of virus introduction into the state of Rio de Janeiro from the North region [5].

## 4. Discussion

The Rio de Janeiro state, in the Southeast region of Brazil, registered 423,084 suspected dengue cases from September 2023 to July 2024. However, only 30% of these cases were confirmed positive for DENV, suggesting the potential silent circulation of other arboviruses. Based on the analysis of ZDC-negative cases, we identified the Middle Paraíba region as one of the areas with the highest negativity rates, coupled with a disproportionately high number of test requests (Figure 1, Supplementary Table S2). As a result, we conducted a retrospective investigation that revealed an outbreak in a small village named Cacaria, located in the municipality of Piraí.

The cluster of cases identified in this study reinforces the spatial transmission dynamics previously described for OROV in other extra-Amazonian states during 2024 [16,17]. The observed spread pattern—predominantly in small, rural municipalities—highlights the need for ecological niches that support viral transmission.

The region most affected in the state of Rio de Janeiro is characterized by a rural area, where banana and coffee cultivation are prevalent [18]. Although associative ecological studies remain preliminary, they have suggested a positive correlation between OROV occurrence and the cultivation of banana, cocoa, oil palm, rubber, coffee, cassava, and sugarcane [19]. These crops tend to produce substantial quantities of decaying organic matter, fostering favorable conditions for the proliferation of *Culicoides paraensis* [20], the primary vector currently identified in OROV transmission [4].

Although OROV spread to other areas of the state of Rio de Janeiro, no evidence of intense viral circulation was observed, and a decline in case numbers occurred after May (Figure 2). The long-distance transmission event was likely mediated by the movement of infected individuals, as illustrated by a concurrent outbreak in Cuba, where viral genomes clustered with Brazilian sequences from 2023 [21]. Subsequently, two imported cases were identified in Italian travelers [22]. The surge in OROV cases across South America and the Caribbean underscores the need to strengthen surveillance systems, particularly in regions with high *Culicoides* vector density.

Phylogenetic analysis revealed that the virus circulating in Rio de Janeiro belongs to the M_1_L_2_S_2_ lineage, the same lineage detected in the Northern region of Brazil from 2022 to 2024 [5]. According to our data, the Middle Paraíba region is the first area with sustained transmission in the Rio de Janeiro state. In 2024, other non-endemic regions such as Bahia, Ceará, Pernambuco, Piauí, Espírito Santo, Minas Gerais, Mato Grosso, Mato Grosso do Sul, and Santa Catarina states also reported the introduction and spread of the M_1_L_2_S_2_ lineage [23,24,25].

Although the data suggest a distinct dispersal capacity for this new lineage, it is essential to consider that the similarity in clinical presentation with other arboviruses, coupled with limited diagnostic capacity in extra-Amazonian regions, may have contributed to OROV’s silent circulation over the years. During epidemic periods, the large number of samples and a lack of tests imply the adoption of selective testing criteria, which can introduce sampling bias. Targeted case selection based on clusters of negativity, combined with ecological determinants, may enhance the effectiveness of surveillance strategies.

Despite the earliest confirmed case being reported in March of 2024 in Piraí, previous peaks of negative tests for dengue in Volta Redonda and Resende cities suggest an even earlier silent circulation in those municipalities, highlighting the importance of further retrospective and continuous active surveillance studies to better understand the dynamics of virus circulation in areas endemic to arboviruses.

## Figures and Tables

**Table 1:** Distribution of Oropouche cases by health region in Rio de Janeiro state, 2023–2024.

Region	Suspected Cases (September 2023 to July 2024)	Suspected Cases (March 2024 to July 2024)	Positive Cases
Coastal Lowland	172	159	2
Ilha Grande Bay	77	75	4
South Center	165	156	2
Metropolitan I	878	804	14
Metropolitan II	240	233	3
Middle Paraíba	1049	448	76
Northwest	190	183	7
North	82	77	0
Mountain	301	293	9
Other state	56	47	1
Total	3210	2475	118

## Data Availability

Data are contained within the article.

## Supplementary Materials

The following supporting information can be downloaded at https://www.mdpi.com/article/10.3390/pathogens14080833/s1, Figure S1: Political map of Rio de Janeiro state by health regions; Table S1: Oropouche virus strains used for phylogenetic analysis; Table S2: Distribution Number and percentage of negative requests examined for Zika, dengue, and chikungunya, from September 2023 to July 2024, by health region, Rio de Janeiro state. Material S1: Identity matrices for analyzed S, M and L sequences.
