# Supplementary material for: Introduction and Spatial–Temporal Distribution of Oropouche Virus in Rio de Janeiro State, Brazil
Source: Pathogens. 2025 Aug 21;14(8):833. doi: 10.3390/pathogens14080833 (PMC12389026; doi:10.3390/pathogens14080833)
Supplement: Supplementary file 1 [file pathogens-14-00833-s001.zip › pathogens-3769809-Supplementary Table S1.pdf]

Supplementary Table S1. Oropouche virus strains used for phylogenetic analysis

Strain	Source of isolation (Host)	Year	Location of isolation	Country of Isolation	GenBank accession no. Segment S	Segment M	Segment L
					S	M	L
TRVL 9760	Human	1955	Sangre Grande	Trinidad and Tobago	KP026181	KP026180	KC759122
AR 19886	*Ochlerotatus serratus* mosquitoes	1960	BR 14 KM 94 (Ipixuna), Pará	Brazil	MG747524	MG747525	HQ830408
AN 19991	*Bradypus tridactylus* sloth	1960	São Miguel do Guamá, Pará	Brazil	KP052852; NC_005777	AF 441119; KP052851; NC_005775	AF 484424; KP052850; NC_005776
NCPV:1409261v	-	1961	Vega de Oropouche	Trinidad and Tobago	MF620127	MF620128	-
H 29086	Human	1961	Belém, Pará	Brazil	MG747527	MG747528	HQ830409
H 29090	Human	1961	Belém, Pará	Brazil	MG747530	MG747531	-
H 121293	Human	1967	Bragança, Pará	Brazil	MG747533	MG747534	HQ830411
AR 136921	*Culex quinquefasciatus* mosquitoes	1968	Belém, Pará	Brazil	MG747536	MG747537	HQ830412
AN 206119	*Bradypus. tridactylus* sloth	1971	Maracanã, Pará	Brazil	MG747539	MG747540	HQ830413
AN 208402	*Bradypus. tridactylus* sloth	1971	Maracanã, Pará	Brazil	MG747542	MG747543	-
AN 208819	*Bradypus. tridactylus* sloth	1971	Maracanã, Pará	Brazil	MG747545	MG747546	-
H 355173	Human	1978	Ananindeua, Pará	Brazil	MG747548	MG747549	HQ830416
AR 366927	*Culicoides paraensis* mosquitoes	1979	Belém, Pará	Brazil	MG747551	MG747552	HQ830417
H 385591	Human	1980	Belém, Pará	Brazil	MG747554	MG747555	HQ830418
H 389865	Human	1980	Manaus, Amazonas	Brazil	MG747506	MG747507	HQ830419
H 390242	Human	1980	Manaus, Amazonas	Brazil	MG747509	MG747510	-
H 472433	Human	1988	Porto Franco, Maranhão	Brazil	MG747512	MG747513	HQ830421
H 472435	Human	1988	Porto Franco, Maranhão	Brazil	MG747515	MG747516	-
H 498913	Human	1990	Machadinho D'Oeste, Rondônia	Brazil	MG747602	MG747603	HQ830423
H 505764	Human	1991	Ariquemes, Rondônia	Brazil	PP357050	PP357049	PP357048
H 505768	Human	1991	Ariquemes, Rondônia	Brazil	MG747605	MG747606	HQ830424
IQT 1690	Human	1992	Iquitos	Peru	KC759127	-	KC759125

H 521086	Human	1993	Barra do Corda, Maranhão	Brazil	MG747518	MG747519	HQ830425
H 532314	Human	1994	Serra Pelada, Pará	Brazil	MG747557	MG747558	HQ830426
H 532422	Human	1994	Serra Pelada, Pará	Brazil	MG747560	MG747561	-
H 532490	Human	1994	Serra Pelada, Pará	Brazil	MG747563	MG747564	-
H 532500	Human	1994	Serra Pelada, Pará	Brazil	MG747566	MG747567	-
H 541140	Human	1994	Altamira, Pará	Brazil	MG747569	MG747570	-
H 543100	Human	1996	Xapuri, Acre	Brazil	MG747503	MG747504	HQ830431
H 543629	Human	1996	Oriximina, Pará	Brazil	MG747572	MG747573	HQ830432
H 543760	Human	1996	Oriximina, Pará	Brazil	MG747575	MG747576	-
H 543857	Human	1996	Oriximina, Pará	Brazil	MG747578	MG747579	-
IQT9924	Human	1999	Iquitos	Peru	-	-	KF697142
AN 622998	*Callitrhix* sp.monkeys	2000	Arinos, Minas Gerais	Brazil	MG747521	MG747522	HQ830436
H 669314	Human	2003	Parauapebas, Pará	Brazil	MG747581	MG747582	HQ830435
H 669315	Human	2003	Parauapebas, Pará	Brazil	MG747584	MG747585	HQ830437
H 682426	Human	2004	Porto de Moz, Pará	Brazil	MG747587	MG747588	-
H 682431	Human	2004	Porto de Moz, Pará	Brazil	MG747590	MG747591	-
H 708139	Human	2006	Magalhães Barata, Pará	Brazil	MG747593	MG747594	HQ830440
H 707287	Human	2006	Magalhães Barata, Pará	Brazil	MG747596	MG747597	-
H 708717	Human	2006	Maracanã, Pará	Brazil	MG747599	MG747600	-
Madre de Dios, FMD 1303	Human	2007	-	Peru	KF697146	KF697145	KF697147
IQE7894	Human	2008	Iquitos,Loreto	Peru	KP795086	KP795085	-
BeH759620	Human	2009	Mazagao, Amapa	Brazil	KP691623	KP691622	-
BeH759621	Human	2009	Mazagao, Amapa	Brazil	KP691608	KP691607	-
BeH759622	Human	2009	Mazagao, Amapa	Brazil	KP691611	KP691610	-
BeH759624	Human	2009	Mazagao, Amapa	Brazil	KP691605	KP691604	-
BeH759625	Human	2009	Mazagao, Amapa	Brazil	KP691614	KP691613	-
BeH759629	Human	2009	Mazagao, Amapa	Brazil	KP691620	KP691619	KP691618
BeH759640	Human	2009	Mazagao, Amapa	Brazil	KP691617	KP691616	-
BeH759646	Human	2009	Mazagao, Amapa	Brazil	KP691632	KP691631	KP691630
Iquitos-MIS-0397	Human	2009	Lima	Peru	KJ866386	KJ866387	KJ866388
Madre de Dios- INHRR 17a-10	Cebus, sp.	2010	Anzoategui	Venezuela	KJ866389	KJ866390	KJ866391

Perdoes BeAn789726	Callitrhix sp. (monkeys)	2012	-	Brazil	KP691626	KP691625	KP691624
Perdoes BeAn790177	Callitrhix sp. (monkeys)	2012	-	Brazil	KP691629	KP691628	-
Haiti-1	Human	2014	-	Haiti	MN264269	MN264268	MN264267
ILMD_TF29	Human			Brazil	PP154170	PP154171	PP154172
D-057/057	Human	2016	Esmeraldas	Ecuador	MK506818	MK506823	-
D-087/058	Human	2016	Esmeraldas	Ecuador	MF926352	MF926353	MF926354
BR_AM_ILMD_0240AOS	Human (urine)	2016	Manaos, Amazonas	Brazil	MN419358	MN419357	MN419356
FCT00025/COL/2017	Human	2017	-	Colombia	MK643115	MK643116	-
Bel90435/H853382	Human	2018	-	Brazil	MT879230	MT879229	MT879228
Saul 17225	Human	2020	-	French Guiana	OL689332	OL689333	OL689334
0200178W	Human	2020	Cucuta	Colombia	OP244879	OP244878	OP244877
LET-352	Human	2021	Leticia	Colombia	OP244882	OP244881	OP244880
LET-882	Human	2021	Leticia	Colombia	OP244885	OP244884	OP244883
Alto Amazonas/Peru/2022	Human	2022	Alto Amazonas	Peru	PP966971	PP966979	PP966987
Roraina/Brazil/2022	Human	2022	PP153982/Roraina/Brazil/2022	Brazil	PP153981	PP153982	PP153983
Rondonia/Brazil/2023	Human	2023	PP153946/Rondonia/Brazil/2023	Brazil	PP153945	PP153946	PP153947
Rondonia/Brazil/2023	Human	2023	PP154018/Rondonia/Brazil/2023	Brazil	PP154017	PP154018	PP154019
Amazonas/Brazil/2023	Human	2023	PP154045/Amazonas/Brazil/2023	Brazil	PP154044	PP154045	PP154046
Amazonas/Brazil/2023	Human	2023	PP154093/Amazonas/Brazil/2023	Brazil	PP154092	PP154093	PP154094
Acre/Brazil/2023	Human	2023	PP154153/Acre/Brazil/2023	Brazil	PP154152	PP154153	PP154154
Maynas/Peru/2023	Human	2023	Maynas	Peru	PP966964		PP966980
Puerto Maldonado/Peru/2023	Human	2023	Puerto Maldonado	Peru	PP966968	PP966976	PP966984
Maynas/Peru/2024	Human	2024	Maynas	Peru	PP966965	PP966973	PP966981
Maynas/Peru/2024	Human	2024	Maynas	Peru	PP966966	PP966974	PP966982
Maynas/Peru/2024	Human	2024	Maynas	Peru	PP966967	PP966975	-
Puerto Maldonado/Peru/2024	Human	2024	Puerto Maldonado	Peru	PP966969	PP966977	PP966985
Puerto Maldonado/Peru/2024	Human	2024	Puerto Maldonado	Peru	PP966970	PP966978	PP966986
-	Human	2024	-	Italy	PP952117	PP952118	PP952119
RJ/087/BRA/2024	Human	2024	Angra dos Reis, Rio de Janeiro (087)	Brazil	PQ295303	-	PQ349278/
RJ/175/BRA/2024	Human	2024	Angra dos Reis,Rio de Janeiro (175)	Brazil	-	PQ349299	-
RJ/238/BRA/2024	Human	2024	Angra dos Reis, Rio de Janeiro (238)	Brazil	-	PQ3439300	PQ349293

RJ/293/BRA/2024	Human	2024	Paracambi, Rio de Janeiro (293)	Brazil	PQ295304	PQ349301	PQ349279
RJ/401/BRA/2024	Human	2024	Nova Friburgo, Rio de Janeiro (401)	Brazil	-	-	PQ349286
RJ/626/BRA/2024	Human	2024	Guapimirim, Rio de Janeiro (626)	Brazil	-	PQ349302	PQ349284
RJ/720/BRA/2024	Human	2024	Valença, Rio de Janeiro (720)	Brazil	PQ295305	PQ295305	PQ349293
RJ/722/BRA/2024	Human	2024	Bom Jesus de Itabapoana, Rio de Janeiro (722)	Brazil		PQ349304	PQ349296
RJ/805/BRA/2024	Human	2024	Saquarema, Rio de Janeiro (805)	Brazil	PQ295306	PQ349305	PQ349291
RJ/817/BRA/2024	Human	2024	Bom Jesus de Itabapoana, Rio de Janeiro (817)	Brazil	PQ295307	-	PQ349283
RJ/845/BRA/2024	Human	2024	Japerí, Rio de Janeiro (845)	Brazil	PQ295308	-	PQ349288
RJ/854/BRA/2024	Human	2024	Guaratiba, Rio de Janeiro (854)	Brazil	-	PQ349306	PQ349281
RJ/868/BRA/2024	Human	2024	Mesquita, Rio de Janeiro (868)	Brazil	PQ295309	-	PQ349282
RJ/883/BRA/2024	Human	2024	Piraí, Rio de Janeiro (883)	Brazil	PQ295310	-	PQ349295
RJ/889/BRA/2024	Human	2024	Pirai, Rio de Janeiro (889)	Brazil	PQ295311	PQ349307	PQ349289
RJ/890/BRA/2024	Human	2024	Pirai, Rio de Janeiro (890)	Brazil	PQ295312	PQ349308	PQ349298
RJ/900/BRA/2024	Human	2024	Volta redonda, Rio de Janeiro (900)	Brazil	PQ295313	PQ349309	PQ349280
RJ/911/BRA/2024	Human	2024	Pirai, Rio de Janeiro (911)	Brazil	PQ295314	PQ349310	PQ349297
RJ/913/BRA/2024	Human	2024	Pirai, Rio de Janeiro (913)	Brazil	PQ295315		PQ349290
RJ/920/BRA/2024	Human	2024	Guapimirim, Rio de Janeiro (920)	Brazil	PQ295316	PQ349312	PQ349287
RJ/986/BRA/2024	Human	2024	Barra Mansa, Rio de Janeiro (986)	Brazil	-	PQ349312	PQ349292
RJ/989/BRA/2024	Human	2024	Guapimirim, Rio de Janeiro (989)	Brazil	-	-	PQ349285
