# Supplementary material for: Introduction and Spatial–Temporal Distribution of Oropouche Virus in Rio de Janeiro State, Brazil
Source: Pathogens. 2025 Aug 21;14(8):833. doi: 10.3390/pathogens14080833 (PMC12389026; doi:10.3390/pathogens14080833)
Supplement: Supplementary file 1 [file pathogens-14-00833-s001.zip › pathogens-3769809-Supplementary Table S2.pdf]

Supplementary Table S2 – Number and percentage of negative requests examined for Zika, dengue, and chikungunya, from September 2023 to July 2024, by health region, Rio de Janeiro state.

Health regions	Pop.	Sep/23		Oct/23		Nov/23		Dec/23		Jan/24		Feb/24		Mar/24		Apr/24		May/24		Jun/24		Jul/24	
		n	%	n	%	n	%	n	%	n	%	n	%	n	%	n	%	n	%	n	%	n	%
Ilha Grande Bay	846,933	99	88.4	105	85.4	87	73.7	115	62.5	381	64.1	683	74.1	364	71.5	64	60.4	36	52.2	26	68.4	35	79.5
Coastal Lowland	253,897	115	78.8	134	84.8	103	67.3	133	55.4	580	50.9	1,361	64.0	806	63.5	305	55.8	138	52.7	46	65.7	28	63.6
South Central Fluminense	320,003	23	88.5	25	96.2	38	79.2	88	60.7	357	55.3	942	61.5	321	44.8	151	44.2	95	55.6	38	70.4	9	56.3
Middle Paraíba	865,130	203	92.7	293	86.7	274	63.3	605	51.8	1,913	59.0	2.429	60.8	820	54.8	633	50.6	384	57.1	158	75.2	55	77.5
Metropolitan I	9,705,577	1.268	87.3	1,188	82.7	1,448	70.1	2,553	66.3	8.482	70.9	11,399	81.4	4,573	76.8	1,833	75.3	860	73.2	516	81.0	530	84.7
Metropolitan II	1,908,751	86	94.5	41	91.1	65	85.5	94	74.6	494	72.1	2,295	84.5	770	80.3	248	76.3	199	68.9	111	80.4	65	79.3
Northwest	324,037	45	95.7	79	92.9	59	88.1	65	82.3	227	57.0	427	67.1	528	71.0	290	73.0	106	70.7	58	81.7	31	81.6
North	920,826	5	83.3	7	46.7	14	82.4	9	56.3	62	41.6	287	53,5	157	64.1	88	51.2	40	49.4	47	54.7	53	76.8
Mountain	910,020	50	98.0	86	97.7	65	85.5	149	70.3	407	52.5	779	61,7	481	46.5	441	48.5	342	52.8	152	76.8	71	83.5
**RJ State**	**16,055,174**	**1894**	**88.1**	**1,958**	**84.6**	**2,153**	**70.5**	**3,811**	**63.3**	**12,903**	**65.9**	**20,602**	**74.3**	**8,820**	**68.2**	**4,053**	**62.5**	**2,200**	**62.6**	**1,152**	**76.7**	**877**	**81.6**
