# Supplementary figures and images for: Introduction and Spatial–Temporal Distribution of Oropouche Virus in Rio de Janeiro State, Brazil
Source: Pathogens. 2025 Aug 21;14(8):833. doi: 10.3390/pathogens14080833 (PMC12389026; doi:10.3390/pathogens14080833)

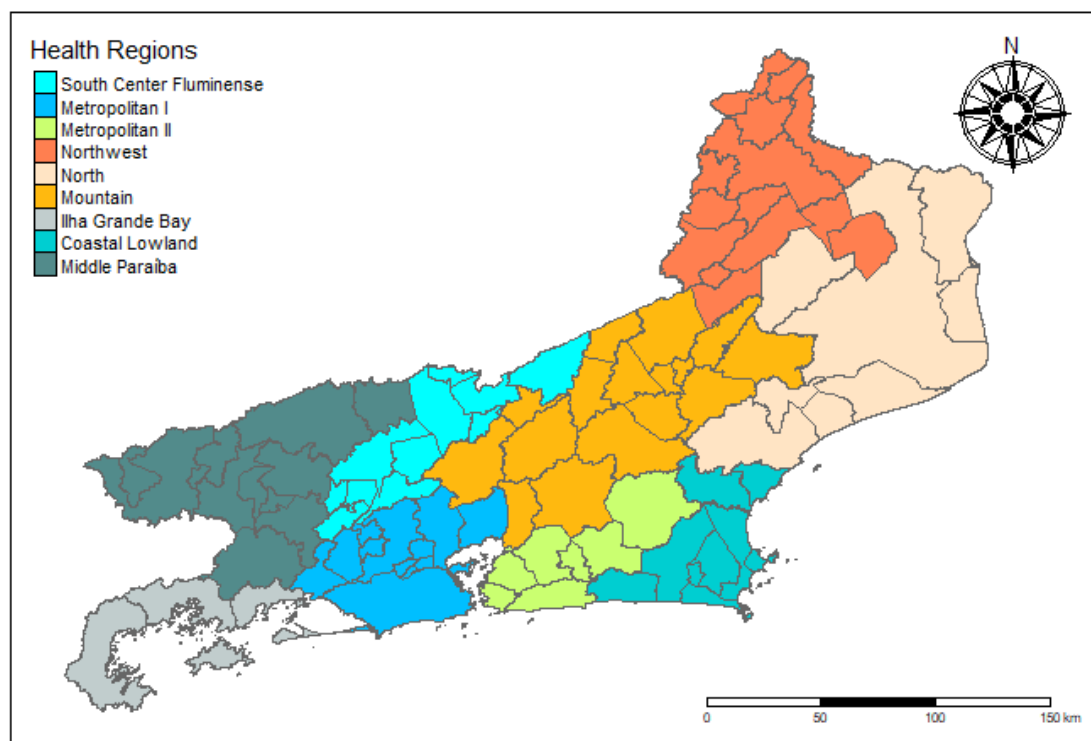

Supplementary Figure S1 – Political map of Rio de Janeiro state by health regions

Supplement: Supplementary file 1 [file pathogens-14-00833-s001.zip › pathogens-3769809-Supplementary Figure S1.pdf]
